# Genetic characterization of influenza A(H3N2) viruses circulating in coastal Kenya, 2009‐2017

**DOI:** 10.1111/irv.12717

**Published:** 2020-01-13

**Authors:** D. Collins Owuor, Joyce M. Ngoi, James R. Otieno, Grieven P. Otieno, Festus M. Nyasimi, Joyce U. Nyiro, Charles N. Agoti, Sandra S. Chaves, D. James Nokes

**Affiliations:** ^1^ Virus Epidemiology and Control Research Group Kenya Medical Research Institute (KEMRI) ‐ Wellcome Trust Research Programme Kilifi Kenya; ^2^ School of Health and Human Sciences Pwani University Kilifi Kenya; ^3^ Influenza Division Centres for Disease Control and Prevention (CDC) Nairobi Kenya; ^4^ School of Life Sciences and Zeeman Institute for Systems Biology and Infectious Disease Epidemiology Research (SBIDER) University of Warwick Coventry UK

**Keywords:** evolution, hemagglutinin, influenza A(H3N2) virus, Kilifi, coastal Kenya, next‐generation sequencing

## Abstract

**Background:**

Influenza viruses evolve rapidly and undergo immune driven selection, especially in the hemagglutinin (HA) protein. We report amino acid changes affecting antigenic epitopes and receptor‐binding sites of A(H3N2) viruses circulating in Kilifi, Kenya, from 2009 to 2017.

**Methods:**

Next‐generation sequencing (NGS) was used to generate A(H3N2) virus genomic data from influenza‐positive specimens collected from hospital admissions and health facility outpatients presenting with acute respiratory illness to health facilities within the Kilifi Health and Demographic Surveillance System. Full‐length HA sequences were utilized to characterize A(H3N2) virus genetic and antigenic changes.

**Results:**

From 186 (90 inpatient and 96 outpatient) influenza A virus‐positive specimens processed, 101 A(H3N2) virus whole genomes were obtained. Among viruses identified in inpatient specimens from 2009 to 2015, divergence of circulating A(H3N2) viruses from the vaccine strains A/Perth/16/2009, A/Texas/50/2012, and A/Switzerland/9715293/2013 formed 6 genetic clades (A/Victoria/208/2009‐like, 3B, 3C, 3C.2a, 4, and 7). Among viruses identified in outpatient specimens from 2015 to 2017, divergence of circulating A(H3N2) viruses from vaccine strain A/Hong Kong/4801/2014 formed clade 3C.2a, subclades 3C.2a2 and 3C.2a3, and subgroup 3C.2a1b. Several amino acid substitutions were associated with the continued genetic evolution of A(H3N2) strains in circulation.

**Conclusions:**

Our results suggest continuing evolution of currently circulating A(H3N2) viruses in Kilifi, coastal Kenya and suggest the need for continuous genetic and antigenic viral surveillance of circulating seasonal influenza viruses with broad geographic representation to facilitate prompt and efficient selection of influenza strains for inclusion in future influenza vaccines.

## 1 INTRODUCTION

Seasonal influenza viruses infect 5%‐15% of the global population annually, resulting in 290 000‐650 000 deaths each year.^1^, ^2^ The disease burden is highest in developing countries especially in sub‐Saharan Africa,^3^, ^4^, ^5^ where influenza viruses may circulate year‐round without clear seasonality; this is in contrast to the clear seasonality observed in temperate climatic regions.^6^ Safe influenza vaccines exist,^1^ but effectiveness depends on host immune responses and how well the vaccine strains match the strains in circulation.^7^ Vaccine effectiveness can be low when there is a mismatch between vaccine selected strains and circulating viruses.^8^

Influenza A viruses (IAV) cause the majority of influenza‐associated disease burden and are further classified into subtypes based on the combination of their hemagglutinin (HA) and neuraminidase (NA) surface glycoproteins.^1^ IAV, especially A(H3N2) virus, evolve rapidly and undergo immune driven selection.^9^ This occurs through changes in viral antigenic epitopes that result in evasion of immune recognition and mainly involves mutations in the HA and NA gene segments.^10^, ^11^

The HA glycoprotein is the primary target of host neutralizing antibodies, which inhibit the binding of HA to sialic acid receptors present on epithelial cell membranes of the upper respiratory tract.^12^ Influenza A(H3N2) virus HA possesses defined antigenic epitopes (five sites designated A through E) and receptor‐binding sites.^13^ Accumulation of mutations at these antigenic sites results in viral escape from the host immune response.^14^, ^15^ These sequence drifts on the HA from accumulated mutations are observed more frequently in A(H3N2) virus than A(H1N1) virus.^8^, ^16^ For example, during the 2013‐14 influenza season, A(H3N2) virus clade 3C.2a viruses possessing a new glycosylation site in antigenic site B of HA emerged and predominated among circulating A(H3N2) viruses which led to a low or null vaccine effectiveness for that season.^17^, ^18^, ^19^, ^20^ As vaccine effectiveness may not be fully explained by antigenic analysis using the hemagglutinin inhibition (HI) assay, the availability of high‐throughput platforms to characterize HA genetic groups, for example, next‐generation sequencing (NGS) techniques, can provide more timely information to evaluate protection afforded by vaccination.

Currently, the government of Kenya is considering recommending annual influenza vaccine for young children.^21^ As an influenza vaccination program is implemented, there will be a need to establish genetic and antigenic viral surveillance which could be used to assess how well the vaccine performs and inform public health decisions on vaccination strategies.^7^

We characterized the genetic changes in A(H3N2) viruses circulating in coastal Kenya using full‐length HA sequences generated through next‐generation sequencing (NGS) from respiratory specimens collected from inpatient and outpatient sentinel surveillance sites in coastal Kenya from 2009 to 2017.

## 2 METHODS

### 2.1 Sample sources and molecular screening

The samples used in this study were collected from health facilities within the Kilifi Health and Demographic Surveillance System (KHDSS) on the coast of Kenya.^22^ Non‐residents and residents of KHDSS presenting to these facilities were included. First, 5304 nasopharyngeal/oropharyngeal (NP/OP) swabs were taken from childhood admissions under the age of 5 years with syndromic severe or very severe pneumonia^23^ collected as part of continuous viral pneumonia surveillance at the Kilifi County Referral Hospital (KCH) from January 2009 through December 2016.^23^ Second, 6254 NP swab samples were taken from outpatients of all ages presenting with acute respiratory illness to selected nine outpatient health facilities spread throughout the KHDSS between December 2015 and March 2017. A comprehensive description of the study area and respiratory disease surveillance at KCH and surrounding outpatient health facilities is available from previous reports.^22^, ^23^, ^24^, ^25^ Samples were stored in viral transport medium (VTM) at −80°C prior to molecular screening and subsequent processing.^24^, ^26^, ^27^

Samples were screened for a range of respiratory viruses, including IAV, using a multiplex (MPX) reverse transcription (RT)‐PCR assay employing Qiagen QuantiFast multiplex RT‐PCR kit (Qiagen),^26^ with epidemiological surveillance results for the period 2007‐12 previously reported.^24^ A real‐time PCR cycle threshold (Ct) of <35.0 was used to define virus‐positive samples.^26^

### 2.2 RNA extraction and multi‐segment real‐time polymerase chain reaction

Viral nucleic acid extraction from IAV positive samples (Ct < 35.0) was performed using the QIAamp Viral RNA Mini Kit (Qiagen). Ribonucleic acid (RNA) was reverse transcribed, and the entire genome of influenza was amplified in a single multi‐segment real‐time polymerase chain reaction (M‐RTPCR) using the Uni/Inf primer set.^28^ The amplification was performed in 25 μL reactions containing 8 μL nuclease‐free water, 12.5 μL 2X RT‐PCR buffer, 0.2 μL Uni12/Inf1 (10 μmol/L), 0.3 μL Uni12/Inf3 (10 μmol/L), 0.5 μL Uni13/Inf1 (10 μmol/L), 0.5 μL SuperScript III One‐Step RT‐PCR with Platinum Taq High Fidelity (Invitrogen), and 3 μL extracted RNA. Thermocycling conditions were as follows: 42°C for 50 minutes, 50°C for 10 minutes, 94°C for 2 minutes; four cycles (94°C for 30 seconds, 43°C for 30 seconds and 68°C for 3 minutes and 50 seconds) followed by 30 cycles of 94°C for 30 seconds, 57°C for 30 seconds, and 68°C for 3 minutes and 30 seconds (with the 3 minutes and 30 seconds for the 68°C extension step increased by 10 seconds per subsequent cycle after cycle 1), and a final extension step at 68°C for 10 minutes. Successful amplification was evaluated by running the products on a 2% Agarose gel and visualized on a UV transilluminator after staining with RedSafe Nucleic Acid Staining solution (iNtRON Biotechnology Inc).

### 2.3 Next‐generation sequencing (NGS) of influenza A virus

Following PCR, the amplicons were purified with 1X AMPure XP beads (Beckman Coulter Inc), quantified with Quant‐iT dsDNA High Sensitivity Assay (Invitrogen), and normalized to 0.2 ng/μL. Indexed paired‐end libraries were generated from 2.5 μL of 0.2 ng/μL amplicon pool using Nextera XT Sample Preparation Kit (Illumina) following the manufacturer's protocol. Amplified libraries were purified using 0.8X AMPure XP beads, quantitated using Quant‐iT dsDNA High Sensitivity Assay (Invitrogen), and evaluated for fragment size in the Agilent 2100 BioAnalyzer System using the Agilent High Sensitivity DNA Kit (Agilent Technologies). Libraries were then diluted to 2 nmol/L in preparation for pooling and denaturation for running on the Illumina MiSeq (Illumina). Pooled libraries were NaOH denatured, diluted to 12.5 pmol/L and sequenced on the Illumina MiSeq using 2 × 250 bp paired‐end reads with the MiSeq v2 500 cycle kit (Illumina). Five percent Phi‐X (Illumina) spike‐in was added to the libraries to increase library diversity by creating a more diverse set of library clusters. Low diversity libraries are common for amplicon pools and occur when a significant number of reads have similar sequences, for example, in amplicon pools.

### 2.4 Bioinformatic analysis

Contiguous nucleotide sequence (contigs) assembly was carried out using the FLU module of the Iterative Refinement Meta‐Assembler (IRMA),^29^ which performs iterative segment‐level read sorting based on Lineage Assignment By Extended Learning (LABEL),^30^ and iteratively refines the references to optimize the final assembly based on the Striped Smith‐Waterman (SSW) algorithm.^31^ IRMA quality control, variant calling and phasing, and assembly pipelines were all implemented in the EDGE Bioinformatics environment^32^ using IRMA default settings. All the sequence data were deposited in the Virus Epidemiology and Control (VEC) research group's Data Repository in Harvard Dataverse under the doi https://dataverse.harvard.edu/ and the Global Initiative on Sharing All Influenza Data (GISAID) EpiFlu^™^ database (https://www.gisaid.org/ ) under the accessions EPI_ISL_393682, EPI_ISL_393684‐393703, EPI_ISL_393705‐393709, EPI_ISL_393711‐393723, EPI_ISL_393725‐393753, EPI_ISL_393936‐393946, EPI_ISL_393949, EPI_ISL_393951‐393953, EPI_ISL_393955‐393956, EPI_ISL_393960, EPI_ISL_393963, EPI_ISL_393965‐393969, EPI_ISL_394051‐394052, EPI_ISL_394107‐394112.

### 2.5 Phylogenetic analysis

Consensus nucleotide sequences of HA gene were aligned and translated into amino acids using MUSCLE program implemented in mega v7.0.26.^33^ The sequences of reference strains of known clades and Northern Hemisphere vaccine strains recommended by WHO included in the phylogenetic analysis were obtained from the GISAID EpiFlu^™^ database (https://www.gisaid.org/). Phylogenetic trees were constructed with maximum likelihood (ML) and bootstrap analysis of 1000 replicates using the GTR + G model implemented in mega 7.0.26. The full‐length HA sequences were used to characterize A(H3N2) strains into genetic clades and subclades according to ECDC guidelines.^34^, ^35^

### 2.6 Prediction of potential glycosylation sites

Potential *N*‐linked glycosylation sites on the HA were determined using the NetNGlyc 1.0 server (http://www.cbs.dtu.dk/services/NetNGlyc/ ), in which a threshold of >0.5 suggests glycosylation.

### 2.7 Ethics

Ethical clearance for the study was granted by the KEMRI‐Scientific and Ethical Review Unit (SERU# 3103) and the University of Warwick Biomedical and Scientific Research Ethics Committee (BSREC# REGO‐2015‐6102). Informed consent was sought and received from the study participants for the study.

## 3 RESULTS

### 3.1 Direct sequencing of IAV from clinical specimens using NGS

**Figure 1:**
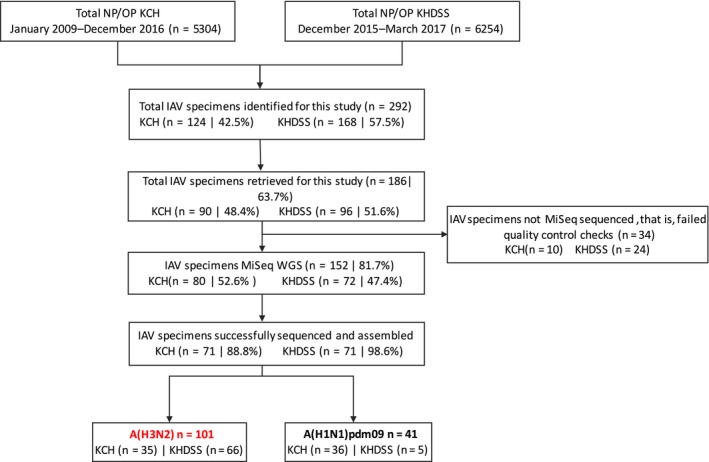
Sample processing flow for KCH inpatient and KHDSS outpatient NP/OP specimens in the Kilifi County surveillance in coastal Kenya, 2009‐17. Sequencing generated 101 and 41 influenza A(H3N2) virus and A(H1N1)pdm09 virus WGS, respectively. One hundred and one A(H3N2) virus full‐length HA sequences were used for this report. KCH, Kilifi County Hospital; KHDSS, Kilifi Health and Demographic Surveillance System; NP/OP, nasopharyngeal/oropharyngeal specimen; MiSeq, Illumina MiSeq sequencer; WGS, whole‐genome sequencing

The specimen processing flow for IAV NGS is shown in Figure 1. A total of 124/5304 (2.3%) and 168/6254 (2.7%) IAV positive specimens were identified from the inpatient and outpatient facilities, respectively. Of the 292 IAV positive specimens identified for this study, 186 (63.7%) were retrieved for this study; the remainder were limited in quantity for RNA extraction, that is, <140 μL. Of these 186 specimens, 152 (81.7%) that passed pre‐sequencing quality control checks were loaded onto the Illumina MiSeq: 80 (52.6%) from the inpatient and 72 (47.4%) from the outpatient studies, respectively, with corresponding success in generating whole‐genome sequences (WGS) for 71 (88.8%) and 71 (98.6%) specimens. A total of 101 (71.1%) A(H3N2) and 41 (28.9) A(H1N1)pdm09 WGS were generated. For this report, 101 determined A(H3N2) virus full‐length HA sequences were used: 35 from the KCH inpatient and 66 from the KHDSS outpatient studies, respectively. The sociodemographic characteristics of these patients are shown in Table 1.

**Table 1:** Demographic and clinical characteristics of KCH inpatients and KHDSS outpatients

	Outpatient		Inpatient	
	n	%	n	%
Age				
0‐11 mo	10	(15)	17	(49)
12‐59 mo	20	(30)	18	(51)
6‐15 y	20	(30)	_	_
16‐64 y	14	(22)	_	_
>=65 y	2	(3)	_	_
Gender				
Female	33	(50)	18	(51)
Male	33	(50)	17	(49)
Cough				
No	2	(3)	0	(0)
Yes	64	(97)	35	(100)
Breathing difficulty				
No	52	(79)	1	(3)
Yes	14	(21)	34	(97)
Indrawing				
No	61	(92)	1	(3)
Yes	5	(8)	34	(97)
Unable to feed				
No	65	(98)	34	(97)
Yes	1	(2)	1	(3)
Oxygen saturation				
<90	1	(2)	5	(14)
>=90	65	(98)	30	(86)
Conscious level				
Alert/Normal	65	(98)	28	(80)
Lethargic	1	(2)	3	(9)
Prostrate	_	_	3	(9)
Unconscious	_	_	1	(2)

Note

Characteristics of 35 KCH inpatients and 66 KHDSS outpatients for whom influenza A(H3N2) HA gene sequences were generated in coastal Kenya, 2009‐17.

Abbreviations: KCH, Kilifi County Referral Hospital; KHDSS, Kilifi Health and Demographic Surveillance System.

### 3.2 Analysis of A(H3N2) virus HA gene sequences

**Figure 2:**
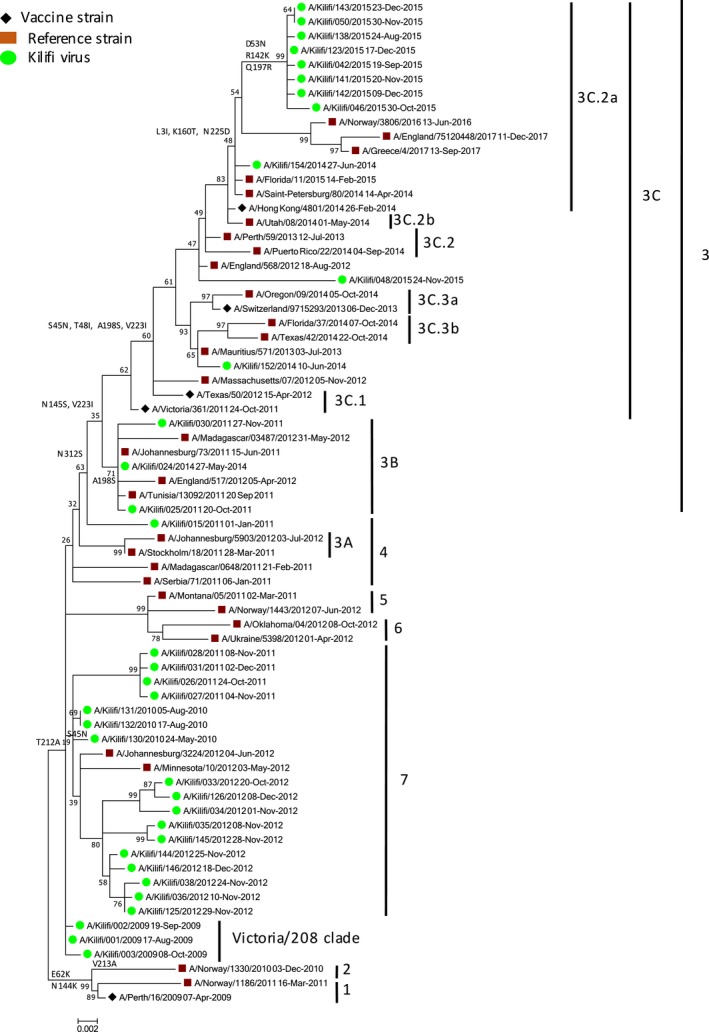
Phylogenetic tree of full‐length HA gene segment for 35 KCH inpatient influenza A(H3N2) virus specimens collected from influenza surveillance in coastal Kenya, 2009‐16. Specimens were collected between January 2009 and December 2016. The phylogenetic tree was inferred from the recommended A(H3N2) vaccine strains (A/Perth/16/2009, A/Victoria/361/2011, A/Texas/50/2012, A/Switzerland/9715292/2013, and A/Hong Kong/4801/2014), selected genetic clade, subclade, and subgroup representative sequences, and inpatient clinical specimens identified in Kilifi, Kenya (2009‐16). The Kilifi strains are color‐coded using clade, subclade, and subgroup while the vaccine strains are coded using a diamond sign. Reference strains and HA2 amino acid substitution numbering are also shown

**Figure 3:**
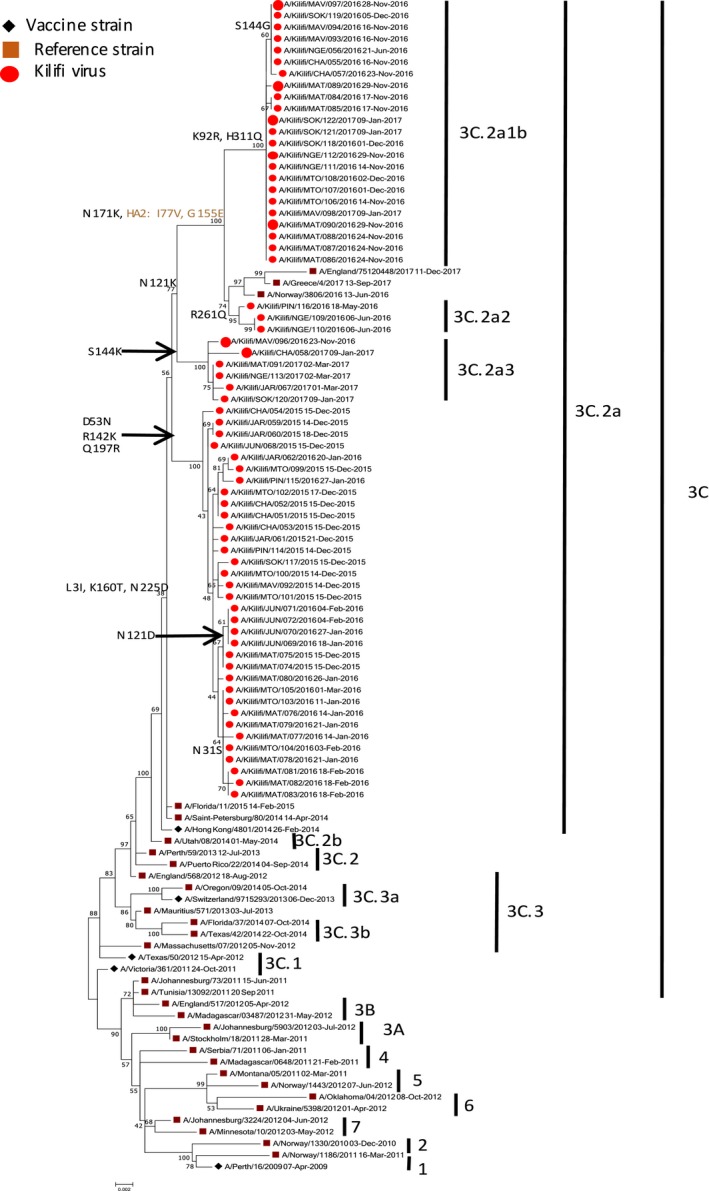
A maximum likelihood phylogenetic tree of the HA gene segment for 66 KHDSS outpatient influenza A(H3N2) virus specimens collected from influenza surveillance in coastal Kenya, 2015‐17. The outpatient specimens were collected between December 2015 and March 2017. The phylogeny was reconstructed from the recommended A(H3N2) vaccine strains (A/Perth/16/2009, A/Victoria/361/2011, A/Texas/50/2012, A/Switzerland/9715292/2013, and A/Hong Kong/4801/2014), genetic clade, subclade, and subgroup sequence representatives, and outpatient clinical specimens identified in Kilifi, Kenya (2015‐17). The Kilifi viruses are color‐coded by clade, subclade and subgroup, while the vaccine strains are coded using a diamond sign. Reference strains and HA2 amino acid substitution numbering are also shown

We investigated the HA gene sequences to characterize the amino acid variations affecting antigenic epitopes, receptor‐binding sites, and potential glycosylation. Full‐length segment coverage of HA was obtained for all the A(H3N2) strains (35 KCH inpatient and 66 KHDSS outpatient) using NGS and sufficient sequence data was available to characterize them into genetic clades and subclades. Among the virus strains identified in hospital inpatient specimens (Figure 2), all the 35 A(H3N2) strains from 2009‐15 diverged from the vaccine strains A/Perth/16/2009 (H3N2)‐like virus, A/Texas/50/2012 (H3N2)‐like virus, and A/Switzerland/9715293/2013 (H3N2)‐like virus. The A(H3N2) strains from 2009 to 2012 (23/35) diverged from the 2009‐12 vaccine strain A/Perth/16/2009 (H3N2)‐like virus and fell into clades A/Victoria/208/2009‐like clade (3/24) and clades 7 (17/24), 4 (1/24), and 3B (2/24), respectively. A virus from 2014 diverged further and fell into the 3B clade (1/35). One virus from 2014 belonging to the 3C clade diverged from the 2014‐15 vaccine strain A/Texas/50/2012 (H3N2)‐like virus (1/35). A virus from 2014 (1/35) and all the 2015 viruses (9/35) belonged to the 3C.2a clade and diverged from the 2015‐16 vaccine strain A/Switzerland/9715293/2013 (H3N2)‐like virus. Among the virus strains identified in the outpatient specimens, the A(H3N2) viruses belonged to genetic clade 3C.2A(34/66), subclades 3C.2a2 (3/66) and 3C.2a3 (6/66), and subgroup 3C.2a1b (23/66) all of which significantly diverged from the 2016‐17 vaccine strain A/Hong Kong/4801/2014 (H3N2)‐like virus (Figure 3).

The high numbers of A(H3N2) viruses in the outpatient setting presented an additional opportunity to investigate the diversity of the virus in a rural community across multiple epidemic seasons. Viruses identified from the outpatient setting were sampled continuously between December 2015 and March 2017. Comparison of the deduced amino acid sequences of all the viruses identified in outpatient specimens to that of the vaccine strain A/Hong Kong/4801/2014 (H3N2)‐like virus, from which all the 2015‐17 strains significantly diverged (Figure 3), showed that the 3C.2a strains (n = 34) differed from the vaccine strain by amino acid variations D53N + R142K + Q197R (HA1). All these strains had additional variations S96N + P194L (HA1). Some 3C.2a strains also possessed additional variations N31S (HA1; n = 10) and N121D (HA1; n = 4). The 3C.2a1b (n = 23) strains were defined by amino acid variations K92R + N121K + N171K + H311Q and I77V + G155E (HA2). Some strains also possessed an additional amino acid variation S144G (HA1; n = 7). The 3C.2a3 strains (n = 6) were defined by amino acid variations N121K + S144K (HA1), whereas the 3C.2a2 strains were defined by N121K + R261Q (HA1) variations. All the 3C.2a, 3C.2a3, 3C.2a2, and 3C.2a1b amino acid substitutions were part of the overall evolution of the strains.

**Figure 4:**
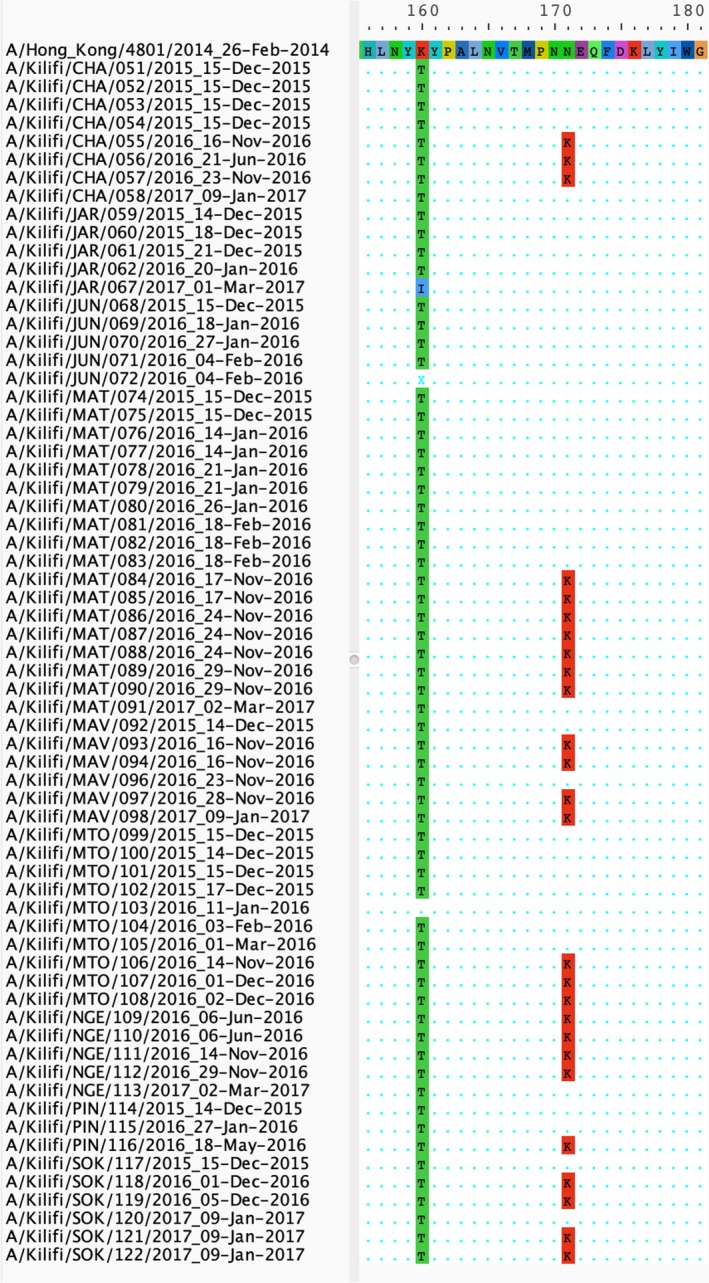
The gain of an *N*‐linked glycosylation site (K160T amino acid substitution) in antigenic site B of HA for 66 KHDSS outpatient A(H3N2) virus specimens collected from influenza surveillance in coastal Kenya, 2015‐17. Multiple sequence alignment of 66 A(H3N2) sequences from Kilifi KHDSS showing the K160T amino acid substitution in 63 of 66 HA gene sequences compared to the 2016‐2017 vaccine strain A/Hong Kong/4801/2014. The K160 amino acid is shown in red whereas the T160 amino acid substitution is shown in green at position 160 of HA1

Importantly, there was gain of an *N*‐linked glycosylation site in antigenic site B of HA due to a K160T amino acid substitution in all but three of the strains identified in the outpatient specimens (63/66; Figure 4). This substitution, which emerged in the 2013‐14 Northern Hemisphere influenza season among clade 3C.2a viruses, results in the glycosylation of residue 158 in antigenic site B of HA that is near the receptor‐binding site and alters the antigenic properties of HA resulting in reduced vaccine effectiveness. This substitution was also observed in all but one of the strains identified in inpatient specimens (8/9) in 2015.

The receptor‐binding site of A(H3N2) virus is highly conserved at amino acids positions 98, 136, 153, 183, 190, 194, 195, and 228 on HA1. However, we did not observe any substitution in these conserved positions in the Kilifi strains.

## 4 DISCUSSION

In this study, we characterized the circulating A(H3N2) viruses in Kilifi between 2009 and 2017 using full‐length HA sequences. The presence of several antigenic site mutations among A(H3N2) virus strains circulating between 2009 and 17 influenza seasons confirms the continuing evolution of circulating strains in Kilifi, Kenya.

Most of the amino acid variations associated with the continuing evolution of A(H3N2) viruses in Kilifi, Kenya have also been reported in other A(H3N2) viruses isolated in Africa, for example, in Cameroon^36^ and Mozambique,^37^ and in Asia, for example, in Thailand.^38^ Thus, the continuing evolution of A(H3N2) in Kilifi is in part due to the global circulation of the virus. Among virus strains identified in inpatient specimens, the 2009‐12 virus strains clustered with the A/Perth/16/2009 (H3N2)‐like clade, while viruses from 2014 clustered with the A/Texas/50/2012 (H3N2)‐like and A/Switzerland/9715293/2013 (H3N2)‐like clades, respectively. Additionally, all the 2015 strains identified from the inpatient specimens clustered with the A/Hong Kong/4801/2014 (H3N2)‐like clade. Among virus strains identified in outpatient specimens between December 2015 and March 2017, the A(H3N2) viruses clustered with the A/Hong Kong/4801/2014 (H3N2)‐like clade.

New antigenic variants emerge when at least one amino acid substitution occurs in the antigenic sites.^39^ The gain or loss of an *N*‐linked glycosylation site in antigenic sites can affect IAV virulence and recognition by neutralizing antibodies.^40^ All but four of the strains circulating in Kilifi, Kenya from 2015 to 17 harbored the K160T amino acid substitution in antigenic site B of HA. This substitution, which results in the gain of an *N*‐linked glycosylation site, is associated with reduced VE for A(H3N2) viruses and has been described previously.^18^ The substitution arose during the 2013‐14 influenza season, and viruses with the substitution predominated among circulating A(H3N2) viruses.^17^ Taken together, these variations underscore the rapid evolution of A(H3N2) viruses in circulation in Kilifi, Kenya.

Kenya is a country where influenza viruses circulate year‐round without clear seasonality.^41^ Additionally, it lies on the equator hence classified as neither a Northern nor a Southern Hemisphere country. Currently, there is no influenza vaccination policy in Kenya, and the number of influenza vaccine doses distributed in the private sector is very limited and based on the Southern Hemisphere vaccine formulations. However, ongoing discussions about introduction of an influenza vaccine recommendation in the government may lead to a formal policy in the near future.^21^ The genetic analysis, as demonstrated in this study, can assist with monitoring the evolution of seasonal influenza viruses. Once influenza vaccination program is implemented in Kenya, monitoring genetic and antigenic changes every year would track the evolution of the seasonal viruses.

Our study had several limitations. The samples used in our studies were limited to the coastal area, yet there may be a wide geographic variation in circulating genetic clades throughout Kenya that we could not capture in our study. Additionally, specific comparison with the Southern Hemisphere vaccine strain composition was limited by the small number of studies reporting on VE in the Southern Hemisphere. These studies, for example, reported moderate VE of influenza vaccine against medically attended and hospitalized influenza during the 2013‐14 influenza season.^42^

## 5 CONCLUSION

The presence of several antigenic site mutations among A(H3N2) strains suggests marked drift of the circulating strains in Kilifi, Kenya. Routine influenza virus surveillance with broad geographic representation can facilitate prompt and efficient selection of influenza strains for inclusion in future influenza vaccines.

## COMPETING INTERESTS

No competing interests were disclosed.

## AUTHORS’ CONTRIBUTION

D. Collins Owuor conceptualized the study, involved in formal analysis, investigated the study, visualized the data, and wrote the original draft of the manuscript. Joyce M. Ngoi investigated the study and validated the study. James R. Otieno involved in formal analysis and visualized the data. Grieven P. Otieno curated the data and involved in formal analysis. Festus M. Nyasimi investigated the study. Joyce U. Nyiro conceptualized the study and visualized the data. Charles N. Agoti conceptualized the study, supervised the study, wrote the original draft of the manuscript, and reviewed and edited the manuscript. Sandra S. Chaves and D. James Nokes conceptualized the study, provided resources, supervised the study, wrote the original draft of the manuscript, and reviewed and edited the manuscript.

## DISCLAIMER

The findings and conclusions in this article are those of the author(s) and do not necessarily represent the official position of the AAS, NEPAD Agency, Wellcome Trust, the UK government, or the USA Centers for Disease Control and Prevention (CDC).
